# The Fecal Microbiome of IBD Patients Is Less Divertible by Bowel Preparation Compared to Healthy Controls: Results From a Prospective Study

**DOI:** 10.1093/ibd/izaf053

**Published:** 2025-04-29

**Authors:** Andreas Blesl, Lukas Binder, Bettina Halwachs, Franziska Baumann-Durchschein, Stefan Fürst, Patrizia Constantini-Kump, Heimo Wenzl, Gregor Gorkiewicz, Christoph Högenauer

**Affiliations:** Division of Gastroenterology and Hepatology, Department of Internal Medicine, Medical University of Graz, Graz, Austria; Division of Gastroenterology and Hepatology, Department of Internal Medicine, Medical University of Graz, Graz, Austria; Division of Gastroenterology and Hepatology, Department of Internal Medicine, Medical University of Graz, Graz, Austria; Institute for Pharmaceutical Sciences, University of Graz, Graz, Austria; Division of Gastroenterology and Hepatology, Department of Internal Medicine, Medical University of Graz, Graz, Austria; Division of Gastroenterology and Hepatology, Department of Internal Medicine, Medical University of Graz, Graz, Austria; Division of Gastroenterology and Hepatology, Department of Internal Medicine, Medical University of Graz, Graz, Austria; Division of Gastroenterology and Hepatology, Department of Internal Medicine, Medical University of Graz, Graz, Austria; Institute of Pathology, Medical University of Graz, Graz, Austria; BioTechMed, Graz, Austria; Division of Gastroenterology and Hepatology, Department of Internal Medicine, Medical University of Graz, Graz, Austria; BioTechMed, Graz, Austria

**Keywords:** inflammatory bowel disease, microbiome, bowel preparation, ulcerative colitis, Crohn’s disease

## Abstract

**Background:**

The fecal microbiome of patients with inflammatory bowel diseases (IBD) is characterized by longitudinal variability. It remains unknown if this is caused by decreased resilience of the microbiome to external factors. We investigated the influence of osmotic diarrhea induced by bowel preparation as a disruptive factor on the fecal microbiome in IBD patients and healthy comparators.

**Methods:**

We conducted a prospective, single-center study including IBD patients and healthy controls scheduled for colonoscopy with uniform bowel preparation. Fecal samples were collected at 7 time points prior, during, and until 3 months after the intervention. 16S rRNA was isolated from stool and sequenced using the Illumina technique.

**Results:**

Twenty-two IBD patients and 17 healthy controls were included in the study. Baseline diversity was higher in healthy controls. Bowel preparation longitudinally decreased alpha diversity and altered beta diversity and taxonomic composition in both groups. Alterations were more pronounced in healthy controls, and the microbial composition converged between the 2 groups. Bowel preparation resulted in an increased relative abundance of *Anaerostipes* and *Coprococcus* in both groups and in decreased relative abundance of *Bifidobacterium* and *Clostridium sensu stricto* in IBD patients and of *Eubacterium hallii* group and *Ruminococcus* in healthy controls. Changes largely restored to baseline composition within 1 week in both groups and remained stable thereafter.

**Conclusions:**

Bowel preparation induced reversible short-term microbial perturbations which were less pronounced in IBD patients than in healthy comparators suggesting that a single external disruptive factor may have less impact on an already altered fecal microbiome.

**Key Messages**

**What is already known?**

The microbiome of inflammatory bowel disease (IBD) patients is characterized by temporal variability. Bowel preparation leads to short-term microbial perturbations in healthy people.

**What is new here?**

The fecal microbiome of IBD patients was less divertible by bowel preparation compared to healthy comparators. Both groups experienced reversible short-term microbial alterations.

**How can this study help patient care?**

Colonoscopy with bowel preparation should not be avoided in IBD patients due to the fear of inducing lasting modifications of the fecal microbiome.

## Introduction

Ulcerative colitis (UC) and Crohn’s disease (CD), denoted as inflammatory bowel diseases (IBD), are immune-mediated chronic inflammatory diseases comprising the gastrointestinal tract. The pathogenesis of these diseases is multifactorial and involves genetic susceptibility, environmental triggers, alterations of the gut microbiome, and defects of the gut barrier. These factors result in mislead responses of the innate and adaptive immune system resulting in chronic intestinal inflammation.^1^

In IBD patients, an increased temporal variability of the fecal microbiome has been reported.^2–4^ Multiple factors including type of diet, smoking, physical activity, stress, disease activity, surgery, travel, and drug intake influence the microbial composition.^5,6^ It is currently unclear whether the variability of the fecal microbiome of IBD patients is the result of accumulation of multiple influencing factors or if it is due to increased susceptibility to alterations in general. Bowel preparation before colonoscopy, which is usually performed by the use of polyethylene glycol (PEG)-based solutions causing osmotic diarrhea, can also disrupt the fecal microbiome and may additionally induce clinical symptoms.^7^ Several studies have examined these changes of the microbiome in healthy subjects.^8–14^ They demonstrated short-term perturbations of diversity and microbial composition.

We conducted the current prospective, investigator-initiated, single-center study to compare the resilience of the fecal microbiome to bowel preparation by a PEG-based solution as a single disruptive factor between IBD patients and healthy controls. We hypothesized that healthy controls will experience short-term quantitative and qualitative perturbations of the microbiome after bowel preparation and that IBD patients will have more prominent and longer lasting changes due to decreased resilience of the microbiome.

## Methods

### Study Population

Between June 2017 and January 2020 patients with IBD, independent of IBD subtype, and healthy controls scheduled for a colonoscopy at the Division of Gastroenterology and Hepatology of the Medical University of Graz, Austria, were eligible for the study.

Inclusion criteria for IBD patients were an established diagnosis of UC or CD by standard clinical criteria^15–17^ and stable medical treatment for at least 3 months prior to study inclusion. Patients with stable disease either in remission or with chronic, mild to moderate disease activity were recruited. Patients with acute flares were excluded due to presumed necessity of changes in medical therapy and an expected change in disease activity.

Inclusion criteria for controls were a scheduled colonoscopy due to post-polypectomy surveillance, a positive test for occult blood, or screening for colorectal cancer in otherwise healthy subjects. Patients with gastrointestinal symptoms were not included.

Exclusion criteria in both groups were prior intestinal resections, treatment with antibiotics, or gastrointestinal infections within 3 months and pregnancy. Intake of laxatives, travel history outside of Europe, and significant changes in dietary habits within 1 month prior to inclusion were also considered as exclusion criteria. Stable intake of proton pump inhibitors (PPIs) and intermittent intake of analgetics were allowed.

During the study period of 3 months, intake of antibiotics, laxatives, and new intake of probiotics were forbidden and all study subjects were asked to keep their previous diet. Adaption of IBD treatment after colonoscopy was allowed if indicated.

### Primary Endpoint

The primary endpoint of the study was to compare alterations of the fecal microbiome between IBD patients and healthy controls in response to osmotic diarrhea induced by bowel preparation.

### Secondary Endpoint

The secondary endpoint was the long-term effect of bowel preparation on the composition of the fecal microbiome.

### Bowel Preparation

All patients received standardized, written instructions for bowel preparation prior to colonoscopy including standard dietary restrictions 3 days before colonoscopy. They received 2 L of Moviprep© (Macrogol [Polyethylenglycol] 3350 13.125 g, Natriumchlorid 0.3507 g, Natriumhydrogencarbonat 0.1785 g, Kaliumchlorid 0.0466 g) in the evening before colonoscopy combined with additional intake of at least 2 L of other fluids.

### Data and Sample Collection

During the study period, 7 fecal samples were collected from each patient: Two samples were collected prior to colonoscopy (taken at 2 different time points: 10 days before and the day before bowel preparation) and served as baseline samples. The third sample was taken during endoscopy (aspiration of colonic fluid). Four follow-up samples were collected 3 and 7 days (early follow-up) and 1 and 3 months (late follow-up) after colonoscopy (Supplementary Figure S1). Fecal samples were stored in collection tubes with DNA stabilizers (Stratec Molecular) according to the manufacturer’s instructions until DNA extraction. Clinical data were obtained from the patient records and with a patient questionnaire. Disease activity in IBD patients was assessed by the Harvey-Bradshaw index^18^ for CD and the partial Mayo score^19^ for UC before colonoscopy and at study termination (month 3). Endoscopic activity in IBD patients was evaluated by the SES-CD (CD)^20^ and the endoscopic Mayo subscore (UC).^19^ An SES-CD score above 6^20^ and an endoscopic Mayo subscore of 2 or 3^19^ confirmed active disease. The quality of bowel preparation was assessed by the Ottawa bowel preparation score^21^ ranging from 0 to 15, with higher scores indicating worse bowel preparation.

### Statistical Analysis

Statistical analysis was done using SPSS Version 26. Patient characteristics were reported as absolute and relative frequencies for categorical data and as medians and interquartile range (q1, q3) for numerical data. Kolmogorov-Smirnov test was used to check for normal distribution. Comparisons between groups were done using *t*-tests, Mann-Whitney *U* tests, Fisher’s exact test, or Chi-square tests as appropriate. All tests were performed on a 5% significance level.

### Microbiome Analysis

#### DNA isolation and 16S amplicon sequencing

Total DNA was isolated by a combination of mechanic and enzymatic lysis with the MagNA Pure LC DNA Isolation Kit III (Bacteria, Fungi; Roche) as described in Klymiuk et al.^22^ Fecal samples in liquid medium with DNA stabilizer (Stratec Molecular Stool Collection Tube, PSP Spin Stool DNA Plus Kit) were vortexed extensively and then 250 µL stool suspension were added to 250 µL 1× PBS and 250 µL Bacterial Lysis Buffer (Roche) and transferred to MagNaLyser green beads (Roche). For mechanical lysis, samples were bead beat 2 times at the MagNaLyser Instrument (Roche) at 6500 rpm for 30 seconds. For enzymatic digestion, samples were first incubated with 25 µL Lysozyme (100 mg/mL) at 37 °C for 30 minutes followed by 43.3 µL Proteinase K (20 mg/mL) at 65 °C for 1 hour. Enzymes were deactivated at 95 °C for 10 minutes followed by a cooling step on ice for 5 minutes. The remaining steps were performed according to instructions from the Magna Pure DNA isolation kit III (Bacteria, Fungi; Roche). One hundred microliters of each sample were used for DNA purification in a MagNaPure instrument (Roche). Total DNA was eluted in 100 µL.

For target-specific PCR amplification of the hypervariable V4 region, the primers 515Fmod (5′-GTGYCAGCMGCCGCGGTAA-3′), Parada et al.,^23^ and 806Rmod (5′-GGACTACNVGGGTWTCTAAT-3′), Appril et al.,^24^ were used in combination with the FastStart High Fidelity PCR system, dNTPack kit (Sigma-Aldrich Chemie). PCR amplification was performed in triplicates in 25 µL reactions containing 2 µL of extracted DNA, 1× Fast Start High Fidelity Buffer, 200 µM dNTPs, 0.4 µM primers, 1.25 U High Fidelity Enzyme, and PCR grade water.

The thermal cycling program was 95 °C for 3 minutes for initial denaturation, followed by 30 cycles of 45 seconds of denaturation at 95 °C, primer annealing at 55 °C for 45 seconds, and extension at 72 °C for 1 minute. Finally, the 72 °C step was extended to 7 minutes and the samples were cooled down to 10 °C. The pooled triplicates were checked on a 1.5% agarose gel, and then normalized using a SequalPrep Normalization Plate (LifeTechnologies) following the manufacturer’s instructions.

About 7.5 µL normalized PCR product was used as template for indexing PCR in a 25 µL reaction for 8 cycles as described in Kozich et al.^25^ for targeted PCR to introduce barcode sequences for each sample. The reagents and the cycling conditions were the same as described above for the target PCR. Five microliters of indexed PCR product from each sample were pooled into the final sequencing library and 30 µL of this pool were loaded to 1.5% agarose gel for purification with the QIAquick Gel Extraction Kit (Qiagen) according to the manufacturer’s instructions. Quantitative and qualitative testing was performed with QuantiFluor ONE dsDNA Dye on a Quantus Fluorometer (Promega) and with a DNA 7500 Kit (Agilent) on a BioAnalyzer 2100 (Agilent). About 6 pM pooled library together with 20% PhiX control DNA (Illumina) were sequenced on a MiSeq desktop sequencer (Illumina) using v3 chemistry and 600 cycles according to the manufacturer’s instructions, resulting in FASTQ raw data files.

#### Computational methods

Demultiplexed raw data were imported and preprocessed using Qiime2.^26^ DADA2 pipeline^27^ of Qiime 2 was used for detecting and correcting raw amplicon sequencing data. Trimming and truncation parameters were chosen according to the interactive quality plot to: trim-left-f/r = 10, trunc-len-f = 282, and trunc-len-r = 206. Sequence variants were classified using the pretrained naïve Bayes classifier (q2-feature-classifier). To increase classification accuracy the classifier was trained by V4 region fragments, extracted according to the used primers, from the 16S rRNA sequences provided by SILVA (version 132, 99% identity^28^). Samples with less than 9512 sequences were excluded from main downstream analysis. In the final step, contingency and contaminant filtering was applied. Only features with a minimum frequency of 10, occur at least in 2 samples, and originate neither from mitochondria nor from chloroplasts were retained.

In total, 3 485 227 sequencing reads from 250 samples with an average count per sample of 13 885 were obtained. Total sum scaling (TSS) was performed. Additionally to Qiime2, analyses were done in Calypso 8.84 (http://cgenome.net/calypso/) or with MicrobiomeAnalyst (https://www.microbiomeanalyst.ca/). For visualization, GraphPad Prism was used. Alpha diversity analysis was performed using observed species and Shannon index on genus level. To outline differences in alpha diversity between groups and time points, analysis of variance (ANOVA) with Bonferroni correction was used. To predict the microbial load from the relative microbial profile, a previously published machine learning approach was applied to the data.^29^ Phylogenetic distances (weighted and unweighted Unifrac) were calculated with Qiime2 and compared with ANOVA and Bonferroni correction as post hoc test. Beta diversity was further displayed with principal component analysis using Bray-Curtis dissimilarity, weighted and unweighted Unifrac distance^30^ on feature level. Permutational multivariate analysis of variance was used as statistic method. To find taxa associated with groups and time points, linear discriminant analysis (LDA) effect size (LEfSe)^31^ was used from phylum to genus levels. Indicated taxa had a logarithmic LDA score > 3.0 and a *P*-value of <.05. Random forests algorithm was used as machine learning method to identify the most important features of LEfSe analysis ranked by their mean decrease accuracy in IBD and controls between baseline 2 and colonoscopy and between baseline samples of both groups.

## Results

### Patient Characteristics

Fifty patients were recruited for the study. There were 11 dropouts: 2 patients had their colonoscopies canceled, 4 patients did not provide follow-up samples after colonoscopy, 4 patients did not provide any sample at all, and 1 patient withdrew from informed consent. All dropouts happened within the first half of the recruitment period. As a reaction to the drop-out rate telephone calls to remind patients to provide samples were introduced. Twenty-two IBD patients and 17 controls completed the study. Healthy controls were older than IBD patients (median [q1, q3]: 54 [53, 61] vs 49 [36, 54] years, *P* < .005). There were no significant differences in gender, intake of medications like PPIs and nonsteroidal anti-inflammatory drugs (NSAIDs), smoking, or the quality of bowel preparation between controls and IBD patients (Table 1).

**Table 1.** Patient characteristics of patients included in the analysis.

	IBD (*n* = 22)	Controls (*n* = 17)	*P*-value
Age (years)	49 (36, 54)	54 (53, 61)	<.005
Female sex	15 (68)	9 (53)	.33
PPI intake	7 (32)	5 (29)	.83
Intermittent NSAID intake	6 (27)	6 (35)	.56
Smoking	3 (14)	3 (18)	.76
Ottawa bowel preparation score	6 (3, 7)	5 (2, 7)	.29

Abbreviations: IBD = inflammatory bowel disease; NSAIDs = nonsteroidal anti-inflammatory drugs; PPI = proton pump inhibitor. Data presented as median (q1, q3) or yes (%).

#### Healthy controls

With 1 exception, controls did not have inflammation upon endoscopy. Five patients had diverticulosis and 8 underwent polypectomy due to tubular adenomas, sessile serrated lesions, or hyperplastic polyps. The maximum number of polyps was 5 in 1 patient, none of these polyps had a size exceeding 10 mm and all were removed with a snare (cold or hot) or with the biopsy forceps if the polyp was 1-3 mm in size or for suspected hyperplastic polyps. In 1 patient minimal ileitis due to NSAIDs intake without histological signs of chronic inflammation was evident.

#### IBD patients

The IBD group consisted of 9 UC and 13 CD patients (Table 2). Eight patients, 1 patient in the UC group and 7 in the CD group, were characterized as having active luminal disease upon colonoscopy. In 2 patients diverticulosis was detected and 1 patient underwent forceps polypectomy of 2 small (<5 mm) hyperplastic polyps. Three patients had their medical therapies changed throughout the study period of 3 months: 1 patient stopped azathioprine, 1 patient started corticosteroid therapy in combination with azathioprine (in both patients the day after colonoscopy), and 1 patient initiated infliximab (1 month after the colonoscopy).

**Table 2.** Patient characteristics of IBD patients included in the analysis.

	Ulcerative colitis (*n* = 9)	Crohn’s disease (*n* = 13)
Age (years)	49 (44, 54)	43 (34, 57)
Female sex	6 (67)	9 (69)
Disease location UC (Montreal classification)		
E1	0 (0)	
E2	6 (67)	
E3	3 (33)	
Disease location CD (Montreal classification)		
L1		2 (15)
L2		6 (46)
L3		2 (15)
L4		3 (23)
Medication at baseline		
Aminosalicylates	6 (67)	3 (23)
*E. coli* Nissle 1917	0 (0)	1 (8)
Thiopurines	4 (44)	3 (23)
Anti-TNF	2 (22)	3 (23)
Vedolizumab	1 (11)	1 (8)
None	0 (0)	4 (31)
Endoscopic score at colonoscopy		
Endoscopic Mayo subscore UC		
0	4 (44)	
1	4 (44)	
2	1 (11)	
3	0 (0)	
Median endoscopic Mayo subscore	1 (0, 1)	
SES-CD		
<3		4 (31)
3-6		2 (15)
7-15		0 (0)
>15		7 (54)
Median SES-CD		10 (0, 20)
Active disease upon colonoscopy	1 (11)	7 (54)
Partial Mayo score at baseline	1 (0, 2)	
Partial Mayo score after 3 months	1 (0, 1)	
Harvey-Bradshaw index at baseline		3 (1, 7)
Harvey-Bradshaw Index after 3 months		1 (0, 2)
Calprotectin (µg/g) at baseline	190 (52, 1000)	241 (93, 1229)
CRP (mg/L) at baseline	2.2 (1.9, 5.7)	4.1 (3, 29)

Abbreviations: CD = Crohn’s disease, CRP = C-reactive protein; IBD = inflammatory bowel disease; UC = ulcerative colitis; disease location was assessed according to the Montreal classification. UC: E1 = proctitis, E2 = left-sided colitis, E3 = pancolitis; CD: L1 = ileal, L2 = colonic, L3 = ileocolonic, L4 = upper gastrointestinal involvement. Data presented as median (q1, q3) or yes (%).

### Changes of the Fecal Microbiome Through Bowel Preparation

#### Alpha diversity

*Short-term decrease of alpha diversity is less pronounced in IBD patients*

**Figure 1.**
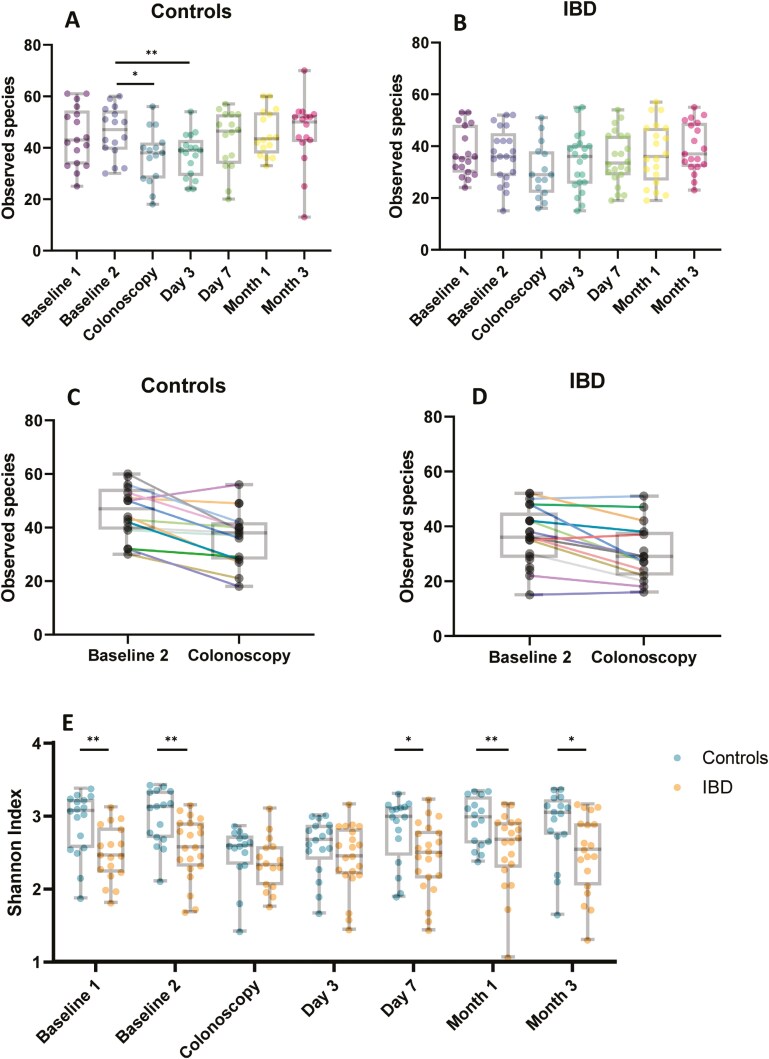
Alpha diversity. (A and B) Alpha diversity of the fecal microbiome at all time points in controls (A) and inflammatory bowel disease (IBD) patients (B) indicated by observed species index and illustrated with box plots. Each dot represents 1 individual. (C and D) Visualization of individual changes of alpha diversity for each proband between baseline 2 and colonoscopy in controls (C) and IBD patients (D) indicated by observed species index. The dots connected by a colored line present 1 person. (E) Alpha diversity indicated by Shannon index comparing controls and IBD patients at each time point illustrated with box plots. Each dot represents 1 individual. Error bars of boxplots range from minimum to maximum, boxes show median ± IQR. Analysis of variance (ANOVA) with Bonferroni correction was used as statistical method. Controls: *n* = 17, IBD: *n* = 22. *P ≤ .05; **P ≤ .01. Abbreviation: IQR = interquartile range.

Baseline samples (baseline 1 and 2) did not show significant differences within groups. Baseline sample 2 was used as comparator to the time points after bowel preparation. Bowel preparation led to a marked and significant decrease in alpha diversity of controls which could still be observed at day 3. Diversity returned to values similar to baseline at day 7 (Supplementary Table S1, Figure 1A and C, Supplementary Figure S2A). In contrast, bowel preparation only resulted in a trend toward decreased alpha diversity in IBD patients on the day of colonoscopy which was not statistically significant (Supplementary Table S1, Figure 1B and D, Supplementary Figure S2B).

*Approximation of alpha diversity in controls and IBD patients through bowel preparation*

Lower alpha diversity in IBD patients compared to controls was evident when comparing baseline samples. The decline of alpha diversity by bowel preparation was more pronounced in controls, leading to an approximation of alpha diversity indices between the 2 groups at the time of colonoscopy and at day 3 (Supplementary Table S1, Figure 1E). At day 7 and after that, alpha diversity in IBD patients was again significantly lower than in controls.

*Predicted microbial load was marginally changed by bowel preparation in both groups*

Fecal microbial load was found significantly decreased at colonoscopy only in IBD patients. Overall, only minor longitudinal changes in the quantitative microbial load were observed (Supplementary Figure S3).

#### Beta diversity

*Phylogenetic distance*

*Changed microbial composition in controls and IBD and decreased phylogenetic dissimilarity between both groups through bowel preparation*

**Figure 2.**
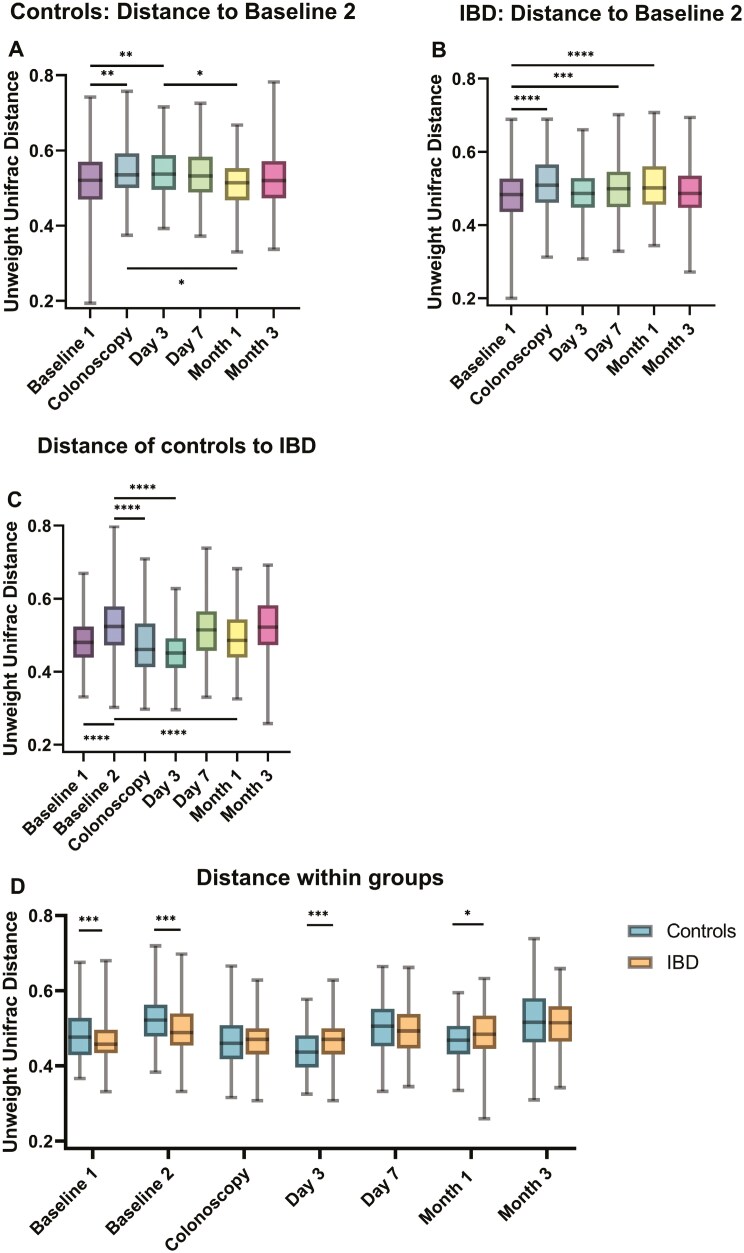
Beta diversity. Unweighted Unifrac distances. (A and B) Distances from all time points to baseline 2 samples of controls (A) and inflammatory bowel disease (IBD) (B). (C) Distance between controls and IBD at all time points. (D) Distance within groups plotted for each time point separately. Distance matrices range from 0 to 1, a lower distance indicates a higher similarity of the microbial community composition. Analysis of variance (ANOVA) with Bonferroni correction was used as statistical method. Error bars of boxplots range from minimum to maximum, boxes show median ± IQR. Controls: *n* = 17, IBD: *n* = 22. *P ≤ .05; **P ≤ .01; ***P ≤ .001; ****P ≤ .0001. Abbreviation: IQR = interquartile range.

Bowel preparation led to longitudinal short-term changes of the fecal microbial composition in each group indicated by increased phylogenetic distances between baseline 2 and colonoscopy samples in controls (unweighted Unifrac) and IBD (unweighted and weighted Unifrac) (Figure 2A and B, Supplementary Table S2). Unweighted Unifrac gives equal importance to rare and common taxa, while weighted Unifrac gives more importance to higher abundant taxa. Prior dissimilarity between both groups decreased with bowel preparation indicated by lower phylogenetic distance at colonoscopy (unweighted and weighted Unifrac) and at day 3 (unweighted Unifrac). Changes were temporally and dissimilarity increased again after bowel preparation within the observation period of 3 months (Figure 2C, Supplementary Table S2).

Dissimilarity of samples within each group at different time points was only marginally influenced by bowel preparation in IBD (decrease of phylogenetic distance at colonoscopy with weighted Unifrac), but in controls microbiome variations within samples decreased with both matrices, most obvious at colonoscopy (weighted Unifrac) and day 3 (unweighted Unifrac). This suggests that bowel preparation harmonizes the microbiome composition of controls (Figure 2D).

*Principal component analysis*

*Lower distraction of beta diversity by bowel preparation in IBD*

**Figure 3.**
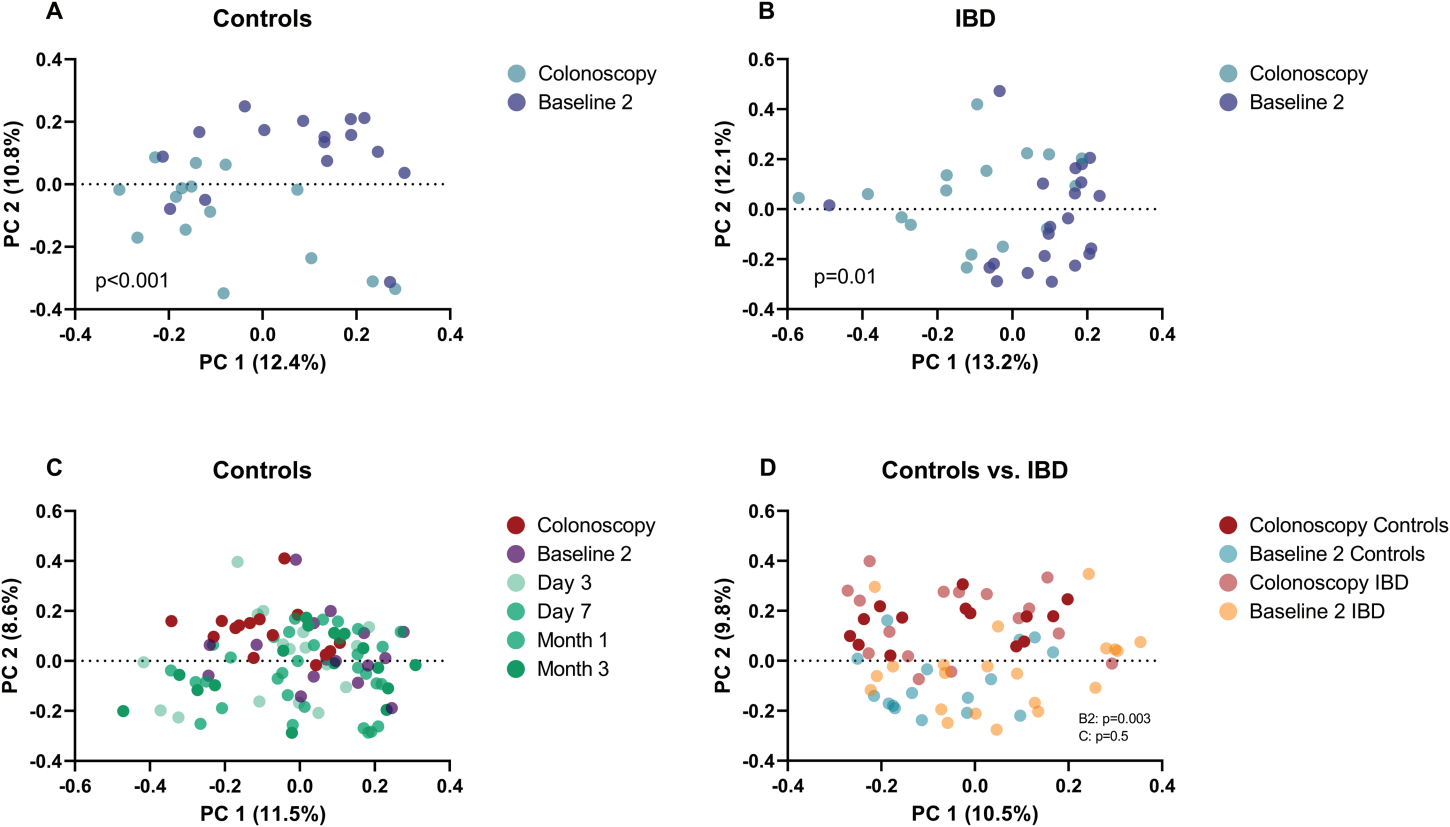
Beta diversity. Principal component analysis (PCoA). (A and B) PCoA plots showing significant short-term changes of beta diversity by bowel preparation (time points: baseline 2, colonoscopy) in controls (A) and inflammatory bowel disease (IBD) patients (B). (C) PCoA plots comparing beta diversity of controls for all time points. (D) Comparisons of controls and IBD at baseline and colonoscopy. Indicated *P*-values show the significance between baseline 2 (B2) and colonoscopy (C) samples between IBD and controls. Shown is Bray-Curtis dissimilarity index. Each dot represents 1 proband. The closer the dots are plotted together, the more similar the composition of the microbiome between samples is. The dotted line represents the zero margin. Permutational MANOVA was used as statistic method. Controls: *n* = 17, IBD: *n* = 22. MANOVA = multivariate analysis of variance.

The 2 baseline samples showed no significant difference in each cohort when analyzed with principal component analysis. Bowel preparation significantly changed the composition of the fecal microbiome in controls and changes lasted until day 3 (Supplementary Table S3, Figure 3A and C). On day 7 and in later samples, microbial changes returned to the initial composition. Bowel preparation also altered beta diversity in IBD patients compared to baseline, but in contrast to controls none of the follow-up samples showed any significant difference compared to baseline (Supplementary Table S3, Figure 3B).

*Approximation of microbiome composition between controls and IBD through bowel preparation*

At baseline, beta diversity differed between controls and IBD (Supplemetary Table S3, Figure 3D). However, bowel preparation led to an approximation of the microbial composition between the 2 groups (Supplementary Table S3, Figure 3D). This lack of separation of composition between controls and IBD patients remained partially evident in follow-up samples after bowel preparation until month 3 using Bray-Curtis and weighted Unifrac metrices (Supplementary Table S3).

#### Taxonomic changes

*Transient alterations of taxonomic composition of the fecal microbiome of controls and IBD patients through bowel preparation*

**Figure 4.**
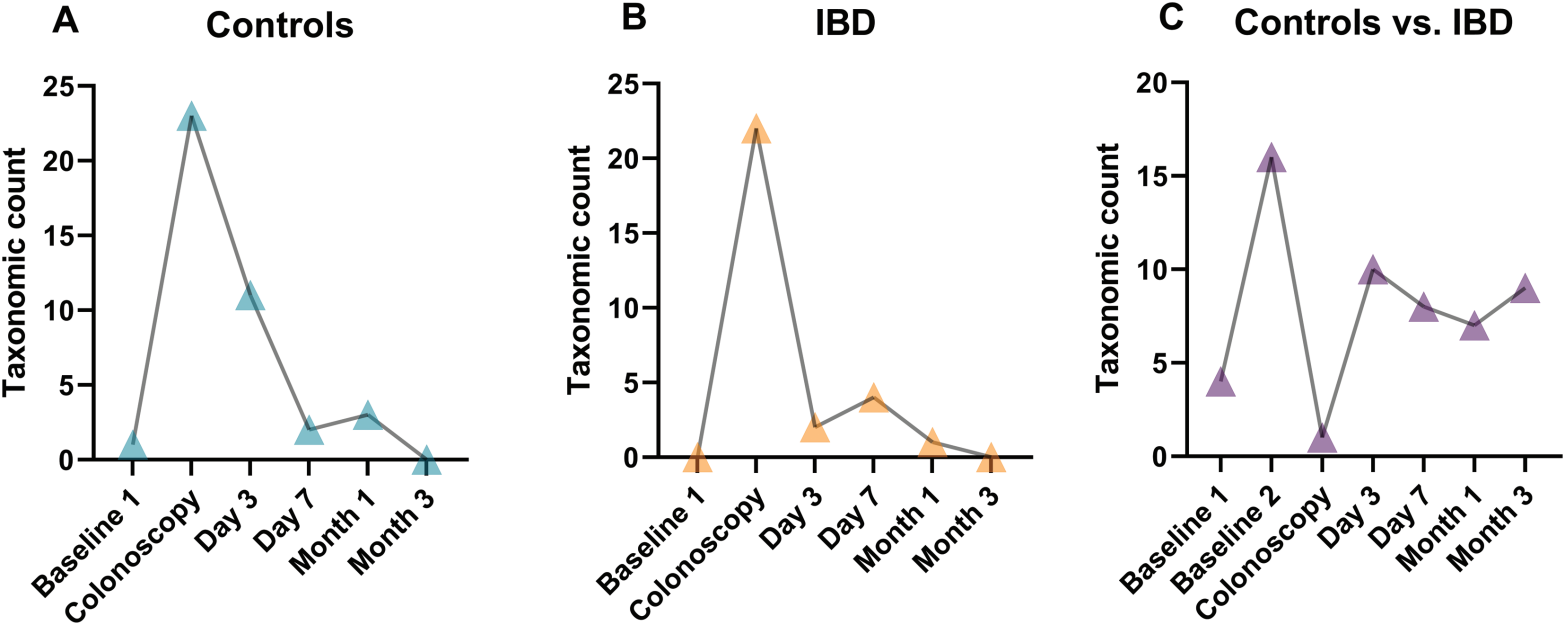
Taxonomic composition. (A and B) Number of distinct taxa from phylum to genus level within groups compared to baseline 2 samples identified with linear discriminant analysis (LDA) effect size (LEfSe) for controls (A) inflammatory bowel disease (IBD) (B). (C) Distinct taxa between controls and IBD at all time points identified with LEfSe. LDA score >3, *P* = .05. Controls: *n* = 17, IBD: *n* = 22.

Taxonomic composition was similar between baseline samples in each group. Bowel preparation led to pronounced taxonomic changes in both cohorts, but these changes disappeared quickly in follow-up samples and were already marginal on day 7 in controls and on day 3 in IBD (Figure 4A and B, Supplementary Figures S4 and S5).

**Figure 5.**
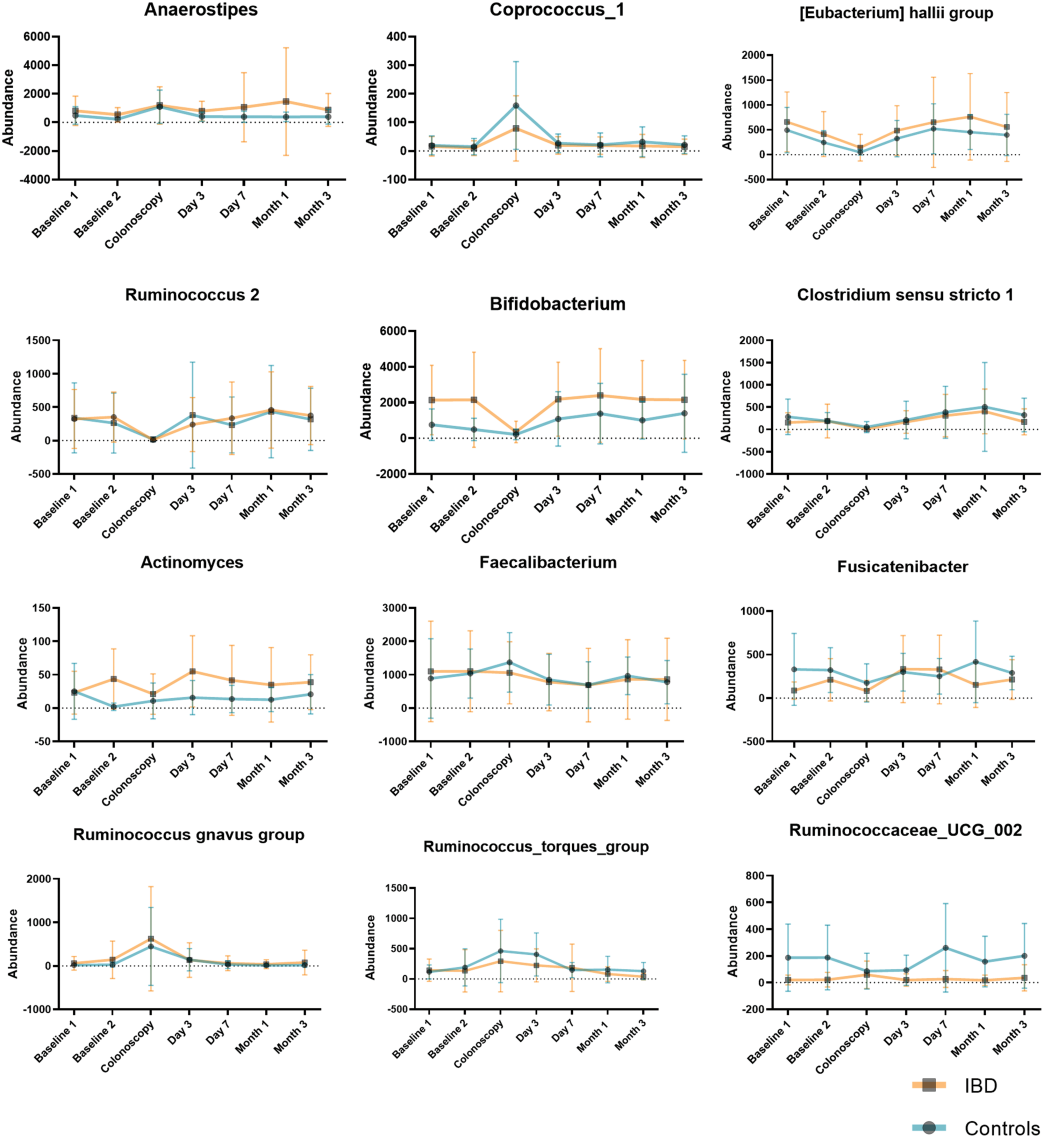
Taxonomic alterations induced by bowel preparation. Displayed genera were identified with Random Forest algorithm as being the most important significant alterations between baseline 2 and colonoscopy in controls and inflammatory bowel disease (IBD) and between baseline 2 samples comparing controls and IBD. Lines show the changes of abundance over all time points indicated as mean with error bars indicating standard deviation. IBD = orange, controls = blue. Controls: *n* = 17, IBD: *n* = 22.

In controls, 23 taxa from phylum to genus level were altered by bowel preparation (Logarithmic LDA score > 3.0; *P* < .05) (Figure 4A, Supplementary Figure S4). The intervention led to the depletion of *Actinobacteria* and to an increase of *Firmicutes* and *Proteobacteria* on phylum level (Supplementary Figure S4). With random forests algorithm the most important altered genera between baseline 2 and colonoscopy were *Anaerostipes* and *Coprococcus_1* (increased at colonoscopy), and *Eubacterium hallii* group and *Ruminococcus_2* (decreased at colonoscopy) (Figure 5).

In IBD, similar as in controls, *Actinobacteria* decreased and *Firmicutes* increased temporally after bowel preparation (Logarithmic LDA score > 3.0; *P* < .05) (Supplementary Figure S5). The most important genera altered by bowel preparation were *Coprococcus_1* (increased at colonoscopy) and *Bifidobacterium* and *Clostridium sensu stricto 1* (decreased at colonoscopy) (Figure 5).

*Loss of differences in microbial composition between controls and IBD through bowel preparation*

The most important genera differentiating controls and IBD at baseline were *Bifidobacterium* and *Actinomyces* (associated with IBD) and *Ruminococcaceae_UCG* and *Fusicatenibacter* (associated with controls) (Figure 5, Supplementary Figure S6). These differences in relative abundance disappeared with bowel preparation, but differences reoccurred from day 3 onwards (Figure 4C, Supplementary Figure S6).

## Discussion

The present prospective study investigated short- and long-term resilience of the fecal microbiome to an external disruptive factor in IBD patients compared to healthy comparators. Our results highlight that bowel preparation induces relevant short-term microbial perturbations in diversity and taxonomic composition, but these alterations are reversible to a large extent and are more pronounced in healthy controls.

Microbes inhabiting the gut seem to play a crucial role in the evolution and progression of IBD and differences in microbial diversity and composition have been demonstrated for IBD patients compared to healthy humans.^32^ In general, the microbiome is stable and regenerates after acute disruptions.^33^ IBD patients have been reported to have increased temporal variability of the intestinal microbiome.^3,4,6^ It might therefore be hypothesized that a volatile intestinal microbiome, as present in IBD patients, is more susceptible to external alterations. To investigate this question, we studied a standardized perturbation of the intestinal microbiome by osmotic diarrhea induced by a PEG-based bowel cleansing protocol for colonoscopy in IBD patients and controls. To our surprise, alterations of the fecal microbiome were more pronounced and longer lasting in healthy controls. After bowel preparation, microbial diversity and composition in controls resembled those of IBD patients, who showed alterations in the microbiome already at baseline. This implies that single external factors, like inducing osmotic diarrhea, may have less impact on an already depleted microbiome as it is present in IBD patients. This is supported by Kumbhari et al. who showed that dysbiosis in IBD seems to adapt to inflammatory conditions, but remains stable after decline of inflammation suggesting a rather stable disturbed community state.^34^

Our data are consistent with prior studies highlighting short-term alterations of alpha and beta diversity through bowel preparation in healthy subjects.^9,11^ The observed increase of *Proteobacteria*, a phylum that comprises many known gastrointestinal pathogens, has also been reported earlier and is further associated with many diseases including IBD.^11,35,36^ We also noticed a reduction of *Actinobacteria*, including the family *Bifidobacteria*, considered as mainly symbiotic species for preserving gut homeostasis.^37^ The depletion of *Actinobacteria* and the increase of *Proteobacteria* observed in this study through bowel preparation therefore mimic changes interpreted as dysbiosis in many intestinal diseases.^35,36^ Existing data about the influence of bowel preparation on the fecal and mucosal microbiome of IBD patients are so far limited to 2 studies. One study with 8 subjects investigated 8 IBD patients at 1 time point pre- and 1 post-bowel preparation.^38^ Decreased alpha diversity after bowel preparation was only detected in the mucosal, but not in the fecal microbiome of IBD patients. These results, although in a different study setting, are therefore widely consistent with our findings. Another study also compared IBD patients to healthy controls, before as well as 3 days and 4 weeks after colonoscopy. The authors observed only minor changes in alpha diversity in UC patients but not in CD or control patients but the study lacks detailed information on short-term longitudinal microbial changes and the fast resaturation of the microbiome.^39^

Our study has the following limitations: Although being the largest study so far evaluating microbial perturbations after bowel preparation, sample size remains restricted. We only investigated the fecal but not the mucosal microbiome as we considered it unethical to perform 6 endoscopic procedures only for study purposes. Furthermore, many of our patients had no or mild activity of their IBD. We excluded IBD patients with severe disease activity because these patients usually require a drastic change in therapy with potential changes of the microbiome which might have biased the results. Although 16S sequencing presents the current standard method for microbiome analysis, it provides less microbial resolution compared to whole metagenome sequencing techniques. Therefore, analysis of composition was only done to genus level. Additionally, 16S rRNA sequencing does not allow quantitative analysis of absolute abundance of taxa.^40^ To overcome this limitation, prediction of the microbial load with a modeling approach was performed.

The strength of our study comprises the carefully performed, prospective study design with data from 2 widely balanced groups with exclusion of other factors influencing the microbiome such as change of therapy or diet. Finally, we analyzed several short- and long-term follow-up samples to investigate microbiome changes over a longer period of time.

## Conclusion

Bowel preparation induced reversible short-term perturbations of the fecal microbiome in controls and IBD patients. A healthy microbiome may be more vulnerable to single external disruptive factors than the primarily already depleted microbiome of IBD patients.

## Supplementary Data

Supplementary data is available at *Inflammatory Bowel Diseases* online.

izaf053_suppl_Supplementary_Material

## Data Availability

The data sets generated and analyzed during the current study are available in the European nucleotide archive (ENA) repository under the Primary accession number PRJEB82596 (or ERA30940459).
