# Supplementary material for: The Fecal Microbiome of IBD Patients Is Less Divertible by Bowel Preparation Compared to Healthy Controls: Results From a Prospective Study
Source: Inflamm Bowel Dis. 2025 Apr 29;31(7):2007–18. doi: 10.1093/ibd/izaf053 (PMC12235133; doi:10.1093/ibd/izaf053)
Supplement: izaf053_suppl_Supplementary_Material [file izaf053_suppl_supplementary_material.docx]

Supplement:


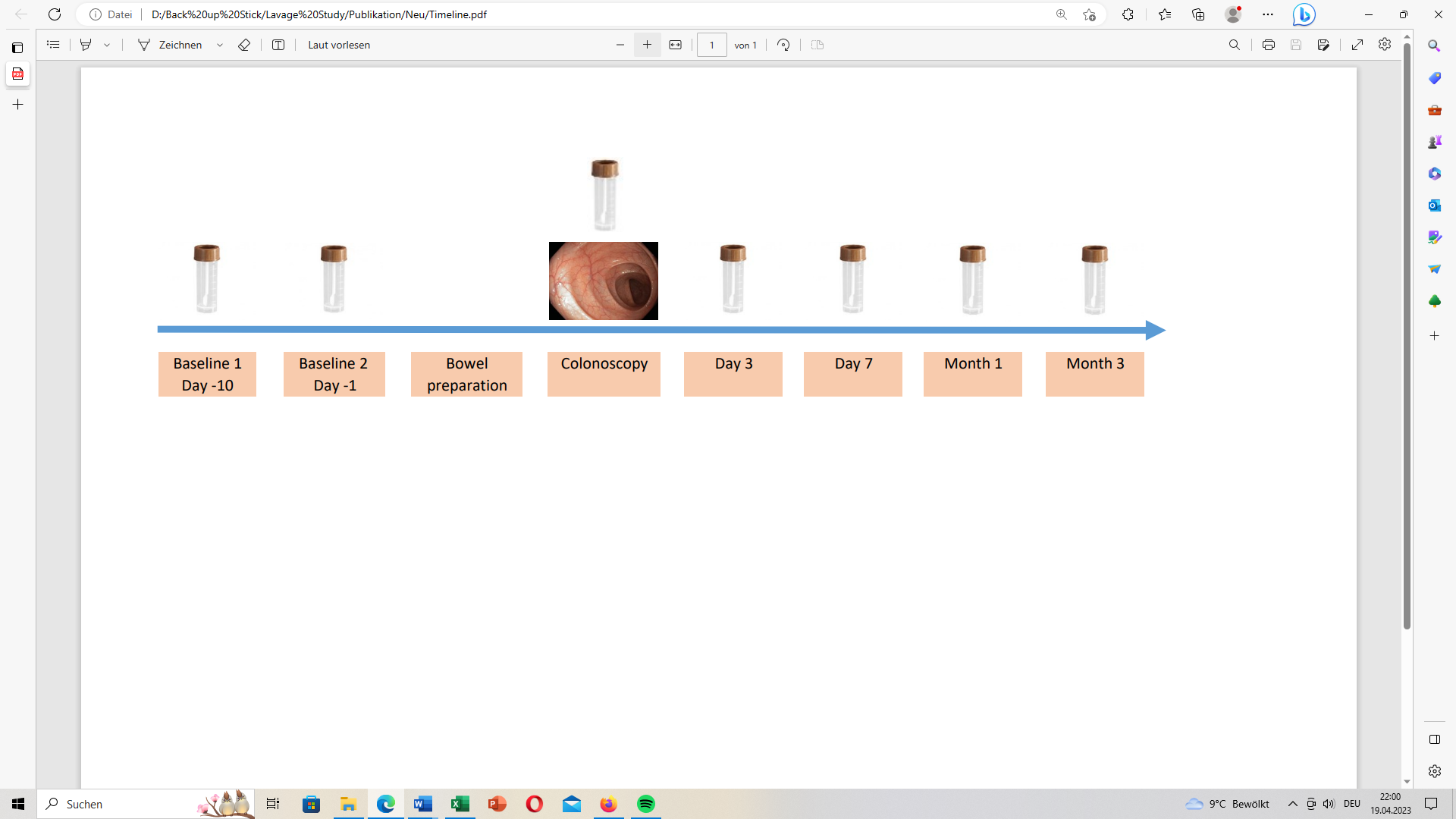


**Supp. Figure 1.** Times of fecal sample collection throughout the study period.

**Supp. Table 1.** **Alpha diversity** examined with observed species and Shannon index highlighted as median (q1, q3) for controls and IBD patients at all time points. Indicated p-values are comparisons of all time points to baseline 2. Row three shows p-values of the comparisons of alpha diversity between controls and IBD patients at each study time point.

|  |  | **Observed species** | **Shannon index** |
| --- | --- | --- | --- |
| **Controls** | Baseline 1 | 43 (34, 55); p=0.7 | 3.0 (2.6, 3.2); p=0.5 |
|  | Baseline 2 | 47 (39, 55); p=NA | 2.8 (2.6, 3.2); p=NA |
|  | Colonoscopy | 38 (28, 42); p=0.02 | 2.6 (2.3, 2.7); p=0.001 |
|  | Day 3 | 39 (29, 43); p=0.009 | 2.6 (2.2, 2.9); p=0.005 |
|  | Day 7 | 47 (34, 53); p=0.4 | 2.9 (2.5, 3.1); p=0.2 |
|  | Month 1 | 44 (38, 54); p=0.9 | 2.9 (2.6, 3.1); p=0.6 |
|  | Month 3 | 50 (42, 53); p=1.0 | 3.0 (2.6, 3.1); p=0.5 |
| **IBD** | Baseline 1 | 36 (30, 48); p=0.7 | 2.5 (2.2, 2.8); p=0.6 |
|  | Baseline 2 | 36 (29, 45); p=NA | 2.6 (2.3, 2.9); p=NA |
|  | Colonoscopy | 29 (22, 38); p=0.1 | 2.3 (2.0, 2.6); p=0.1 |
|  | Day 3 | 36 (26,41); p=0.4 | 2.4 (2.2, 2.8); p=0.3 |
|  | Day 7 | 34 (29, 44); p=0.7 | 2.5 (2.1, 2.8); p=0.4 |
|  | Month 1 | 36 (27,47); p=0.7 | 2.7 (2.3, 2.9); p=0.9 |
|  | Month 3 | 37 (32, 49); p=0.4 | 2.5 (2.0, 2.9); p=0.7 |
| **Controls vs. IBD** | Baseline 1 | p=0.05 | p=0.005 |
|  | Baseline 2 | p=0.007 | p=0.003 |
|  | Colonoscopy | p=0.2 | p=0.3 |
|  | Day 3 | p=0.3 | p=0.2 |
|  | Day 7 | p=0.04 | p=0.02 |
|  | Month 1 | p=0.02 | p=0.01 |
|  | Month 3 | p=0.1 | p=0.04 |

IBD = inflammatory bowel disease

**A**

**B**

**Supp. Figure 2. Alpha diversity.** Individual longitudinal changes of alpha diversity of the fecal microbiome over the study period in controls **(A)** and IBD patients **(B)**. Each line represents one individual. Error bars of boxplots range from minimum to maximum, boxes show median +/- IQR. Alpha Diversity is indicated by observed species index. Controls: n=17, IBD: n=22.

**Supp. Figure 3.** **Predicted microbial load.** Microbial load was estimated based on the taxonomic composition using a machine-learning approach. Plots show range from minimum to maximum, middle lines represent the median. Controls: n=17, IBD: n=22. * p ≤ 0.05.

**Supp. Table 2. Beta diversity. Weighted and unweighted distance matrices.** Distance matrices at all time points to baseline 2 samples in controls and IBD (first two rows). Distance matrices range from 0 to 1, a lower distance indicates a higher similarity of the microbial community composition. Row three shows the phylogenetic dissimilarity between controls and IBD patients at all time points. Data presented as median (q1, q3).

|  |  | **Weighted Unifrac distance** | **Unweighted Unifrac distance** |
| --- | --- | --- | --- |
| **Controls – Distance to baseline 2** | Baseline 1 | 0.21 (0.17, 0.25) | 0.52 (0.47, 0.57) |
|  | Colonoscopy | 0.19 (0.17, 0.23) | 0.54 (0.50, 0.59) |
|  | Day 3 | 0.21 (0.17, 0.30) | 0.54 (0.50, 0.59) |
|  | Day 7 | 0.20 (0.17, 0.26) | 0.53 (0.49, 0.58) |
|  | Month 1 | 0.19 (0.16, 0.24) | 0.51 (0.47, 0.55) |
|  | Month 3 | 0.22 (0.16, 0.26) | 0.52 (0.47, 0.57) |
| **IBD - Distance to baseline 2** | Baseline 1 | 0.20 (0.17, 0.24) | 0.48 (0.44, 0.53) |
|  | Colonoscopy | 0.22 (0.18, 0.26) | 0.51 (0.46, 0.57) |
|  | Day 3 | 0.20 (0.17, 0.24) | 0.49 (0.45, 0.53) |
|  | Day 7 | 0.21 (0.18, 0.26) | 0.50 (0.45, 0.55) |
|  | Month 1 | 0.22 (0.18, 0.26) | 0.50 (0.46, 0.56) |
|  | Month 3 | 0.20 (0.17, 0.25) | 0.49 (0.45, 0.53) |
| **Distance of controls to IBD** | Baseline 1 | 0.22 (0.18, 0.26) | 0.48 (0.44, 0.52) |
|  | Baseline 2 | 0.21 (0.17, 0.24) | 0.52 (0.47, 0.57) |
|  | Colonoscopy | 0.17 (0.13, 0.21) | 0.46 (0.41, 0.53) |
|  | Day 3 | 0.20 (0.15, 0.26) | 0.45 (0.41, 0.49) |
|  | Day 7 | 0.21 (0.17, 0.26) | 0.51 (0.46, 0.57) |
|  | Month 1 | 0.21 (0.17, 0.25) | 0.49 (0.44, 0.54) |
|  | Month 3 | 0.21 (0.16, 0.25) | 0.52 (0.47, 0.58) |

**Supp. Table 3. Beta diversity. Principal component analysis.** Comparisons of beta diversity at all time points to baseline 2 samples in controls and IBD (first two rows) with principal component analysis on feature level. Shown are calculated p-values with permutational MANOVA. Row three shows p-values of the comparisons between controls and IBD patients at all time points. Indicated indices are Bray Curtis, Weighted and Unweighted Unifrac.

|  |  | **Bray Curtis** | **Weighted Unifrac** | **Unweighted Unifrac** |
| --- | --- | --- | --- | --- |
| **Controls** | Baseline 1 | p=1.0 | p=0.9 | p=1.0 |
|  | Colonoscopy | p<0.001 | p<0.001 | p=0.003 |
|  | Day 3 | p=0.1 | p=0.02 | p=0.002 |
|  | Day 7 | p=0.9 | p=0.5 | p=1.0 |
|  | Month 1 | p=1.0 | p=0.9 | p=0.9 |
|  | Month 3 | p=1.0 | p=0.6 | p=1.0 |
| **IBD** | Baseline 1 | p=1.0 | p=0.7 | p=1.0 |
|  | Colonoscopy | p=0.01 | p=0.08 | p=0.03 |
|  | Day 3 | p=0.8 | p=0.2 | p=0.5 |
|  | Day 7 | p=1.0 | p=0.6 | p=0.9 |
|  | Month 1 | p=1.0 | p=0.9 | p=0.9 |
|  | Month 3 | p=1.0 | p=0.9 | p=0.9 |
| **Controls vs. IBD** | Baseline 1 | p=0.007 | p=0.08 | p=0.04 |
|  | Baseline 2 | p=0.003 | p=0.01 | p=0.01 |
|  | Colonoscopy | p=0.5 | p=0.6 | p=0.04 |
|  | Day 3 | p=0.05 | p=0.3 | p=0.7 |
|  | Day 7 | p=0.2 | p=0.3 | p=0.02 |
|  | Month 1 | p=0.2 | p=0.2 | p=0.005 |
|  | Month 3 | p=0.1 | p=0.2 | p=0.04 |

IBD = inflammatory bowel disease

**Supp. Figure 4. Taxonomic differences in controls.** Taxonomic differences from phylum to genus level (Logarithmic LDA score > 3.0; p<0.05) in controls between baseline 2 samples and all other time points assessed with LEfSe analysis. Taxa more prevalent at baseline 2 are coloured in violet, taxa more prevalent at the other time points in blue. No significant findings between baseline 2 and month 3 could be observed. Controls: n=17, IBD: n=22.

**Supp. Figure 5.** **Taxonomic differences in IBD patients.** Taxonomic differences from phylum to genus level (Logarithmic LDA score > 3.0; p<0.05) in IBD between baseline 2 samples and all other time points assessed with LEfSe analysis. Taxa more prevalent at baseline 2 are coloured in violet, taxa more prevalent at the other time points in blue. No significant findings could be observed for the comparison of baseline 2 samples with baseline 1 and month 3. Controls: n=17, IBD: n=22.

**Supp. Figure 6.** **Taxonomic composition comparing controls and IBD patients.** Taxonomic differences from phylum to genus level (Logarithmic LDA score > 3.0; p<0.05) between controls and IBD at all time points assessed with LEfSe analysis. Taxa more prevalent in controls are coloured blue, taxa more prevalent in IBD patients in orange. Controls: n=17, IBD: n=22.
